# Elevated frequencies of total and MAIT cell subsets in patients with knee osteoarthritis

**DOI:** 10.7717/peerj.7443

**Published:** 2019-08-05

**Authors:** Ding Zhao, Wei Zhong, Dongfeng Han, Yingbo Li, Yanfang Jiang, Guishan Gu

**Affiliations:** 1 Department of Orthopedics, First Hospital, Jilin University, Changchun, Jilin, China; 2 Department of Rheumatology, the First Hospital of Qiqihaer, Qiqihaer, Heilongjiang, China; 3 Department of Emergency Medicine, First Hospital, Jilin University, Changchun, Jilin, China; 4 Central Laboratory, First Hospital, Jilin University, Changchun, Jilin, China; 5 Genetic Diagnosis Center, First Hospital, Jilin University, Changchun, Jilin, China

**Keywords:** Osteoarthritis, MAIT cells, Cd8αα, Knee osteoarthritis, Multi-joint impairment

## Abstract

**Background:**

Osteoarthritis (OA) is characterized by the degeneration of joint cartilage, with concomitant changes in the synovium and subchondral bone. Recently, the inflammatory response and involvement of several types of T-cells has been implicated in the development of OA. This study investigated the frequency of MR1-restricted mucosal-associated invariant T (MAIT) cells in patients with knee OA.

**Methods:**

Forty-five patients recently diagnosed with knee OA and 21 age- and gender-matched healthy controls were recruited for this study. Percentages of circulating MAIT cells were assessed by flow cytometry. Plasma cytokine levels were measured using cytometric bead arrays. Associations between the percentages of MAIT cells, plasma cytokine levels, and clinical parameters of OA (erythrocyte sedimentation rate [ESR] and the Western Ontario and McMaster Universities Osteoarthritis Index [WOMAC]) were analyzed using the Spearman correlation test.

**Results:**

The percentages of total, CD8αα, and CD8αβ MAIT cells were higher in patients with OA compared to healthy controls. The percentages of total and CD8αα MAIT cells were higher in patients with multi-joint OA (MOA) compared to patients with knee-only OA (KOA). Plasma IFN-γ and TNF-α levels were elevated in patients with OA compared to healthy controls, and there was a positive correlation between plasma IFN-γ levels and the percentages of total, CD8αα, and CD8αβ MAIT cells. Plasma IFN-γ and IL-17 levels were higher in patients with MOA compared to healthy controls or patients with KOA. There were positive correlations between the percentages of total and CD8αα MAIT cells and clinical parameters (ESR and WOMAC scores) in patients with OA or MOA. Binary logistic regression analysis shown the frequency of MAIT cells was associated with the risk of OA.

**Conclusions:**

MAIT cells and their subpopulations were significantly increased in patients with OA and have potential as biological markers of OA disease severity, especially in patients with MOA.

## Introduction

Osteoarthritis (OA) impairs one or more synovial joints, including weight-bearing joints, such as the knee and the hip joints, and small joints such as those in the hand. Globally, an estimated 3.8% of the population suffers from OA (Martel-Pelletier et al., 2016). The knee joint is most commonly affected, and OA of the knee is a leading cause of pain, morbidity, and disability in OA patients (Martel-Pelletier et al., 2016).

OA is characterized by the degeneration of joint cartilage, with concomitant changes in the synovium and subchondral bone. The etiology of OA is controversial, but it may be associated with factors such as age, sex, obesity, and diet, as well as injury, malalignment, and abnormal loading of the affected joints (Zhang & Jordan, 2010; Li et al., 2017). Accumulating evidence suggests that the pathogenesis of OA in certain patients involves local joint inflammation, which is characterized by the infiltration of inflammatory CD4^+^ and CD8^+^T-cells in the synovial membranes (Berenbaum, 2013; Penatti et al., 2017; De Lange-Brokaar et al., 2012; Haynes, Hume & Smith, 2002; Ponchel et al., 2015; Sakkas & Platsoucas, 2007). More recently, a systemic inflammatory response has been implicated in the development of OA (Qi et al., 2016; Sakkas & Platsoucas, 2007; Symons et al., 1991). A strong connection has been established between OA and several types of T-cells, including Th1, Th2, Th9, Th17, Th22, Tregs, follicular helper T-cells, and cytotoxic T-cells (Li et al., 2017). However, reports describing the role of unconventional T-cells, such as CD1-restricted T-cells, γδ T-cells, major histocompatibility complex (MHC) class Ib-reactive T-cells, and MR1-restricted mucosal-associated invariant T (MAIT) cells, in OA are scarce.

MAIT cells are innate-like T-cells that are widely distributed in blood, mucosal tissues, the liver, and in most joints (Hinks, 2016). In humans, MAIT cells express Vα7.2-Jα33, a semi-invariant T-cell receptor (TCR) α chain, preferentially paired with Vβ2 or Vβ13.2 chains (Treiner et al., 2003; Walker et al., 2012). Three subsets of MAIT cells have been identified, including CD8αα, CD8αβ, and double-negative (DN) MAIT cells, although the functional significance of these subsets is unknown (Hinks, 2016). MAIT cells are restricted by the MHC-related protein MR1 and can recognize and respond to microbially derived riboflavin (Vitamin B2) derivatives (Kjer-Nielsen et al., 2012; Patel et al., 2013). Recently, a minor subset of MAIT cells was shown to detect folate (Vitamin B9)-based antigens or drug metabolites and drug-like molecules presented by MR1, including 6-formylpterin and diclofenac (Gherardin et al., 2016; Keller et al., 2017). Previous studies have successfully identified MAIT cells in the human TCRγδ^−^CD3^+^ cell population using CD161 or IL-18Rα monoclonal antibodies conjugated with a Vα7.2 monoclonal antibody (Chen et al., 2019; Mendy et al., 2019; Paquin-Proulx et al., 2018). When activated, MAIT cells secrete various cytokines, including IFN-γ, TNF-α, and IL-17A (Dusseaux et al., 2011; Kawachi et al., 2006), suggesting their involvement in microbial infections and other inflammatory processes (Kumar & Ahmad, 2017). Accordingly, recent studies have suggested a correlation between MAIT cells and inflammatory diseases, including multiple sclerosis (Annibali et al., 2011; Illes et al., 2004), inflammatory bowel disease (IBD) (Haga et al., 2016; Serriari et al., 2014), type II diabetes, asthma (Hinks, 2016), and rheumatoid arthritis (RA) (Cho et al., 2014). At present, the role of MAIT cells in OA remains to be elucidated.

The objective of this study was to compare the percentages of total, CD8αα, and CD8αβ MAIT cells and the plasma IFN-γ, TNF-α, and IL-17 levels between OA patients and healthy controls. The percentage of MAIT cells and the plasma IFN-γ, TNF-α, and IL-17 levels were correlated with clinical parameters and OA disease severity. Findings from this study will enhance our understanding of the functional significance of MAIT cells in OA.

## Materials & Methods

### Study subjects

Patients with newly diagnosed knee OA admitted to the in-patient department of the First Hospital of Jilin University, China between January 2016 and November 2017 were eligible for this study. Inclusion criteria were: (1) confirmed diagnosis of knee OA according to American College of Rheumatology (ACR) clinical and radiographic criteria (Altman et al., 1986); and (2) no prior history of OA treatment. Exclusion criteria were: (1) history of systemic lupus erythematosus, RA, other autoimmune disorders, cancer, traumatic arthritis, multiple sclerosis, diabetes, immunodeficiency, hypertension, cardiovascular disease, renal failure, gastrointestinal bleeding, depression, chronic inflammatory diseases, or recent infection; and (2) patients who had received glucocorticoid, non-steroidal anti-inflammatory drugs, or immune suppressive treatment during the prior six months. Included patients with knee OA were categorized into two subgroups: knee-only OA (KOA), defined as patients with symptomatic OA localized to one or both knees only, and multi-joint OA (MOA), defined as patients with symptomatic OA of the knees in addition to other joints, such as the hands, hip, or spine (McAlindon et al., 2014). Ethnicity-, age-, and gender-matched healthy volunteers were recruited from the Physical Examination Center of the hospital’s outpatient department as controls. The study protocol was established according to the guidelines of the Declaration of Helsinki and approved by the Human Ethics Committee of Jilin University (approval number: 2015-252). Study subjects provided written informed consent.

### Demographic and clinical characteristics

Study subjects’ demographic characteristics, including age, gender, and BMI were extracted from the medical records. Study subjects’ clinical characteristics, including complete blood cell (CBC) counts, erythrocyte sedimentation rate (ESR), and plasma C-reactive protein (CRP) levels were measured during routine laboratory tests. Knee radiographs were evaluated according to the Kellgren-Lawrence (KL) classification criteria (Kellgren & Lawrence, 1957). OA disease severity was assessed using the Western Ontario and McMaster Universities Osteoarthritis Index (WOMAC), which is a 24-item questionnaire to assess pain, stiffness, and the ability to perform normal daily activities (Bellamy et al., 1988). Items are scored on the 4-point Likert Scale (0 = none, 1 = slight, 2 = moderate, 3 = severe, 4 = extreme), with higher scores representing higher levels of pain, stiffness, and functional impairment.

### Isolation of peripheral blood mononuclear cells (PBMCs)

After an overnight fast, each participant provided a peripheral blood (six mL) sample. PBMCs were isolated by density-gradient centrifugation at 800 g for 30 min using the Ficoll-Paque Plus method (Amersham Biosciences, Buckinghamshire, UK) and resuspended at a concentration of 1 × 10^6^ cells per mL in RPMI-1640 culture medium (Invitrogen, Carlsbad, CA, USA) containing 10% fetal calf serum.

### Flow cytometry analysis

Isolated PBMCs were stained in duplicate with FITC-anti-CD3 (BD Biosciences, San Diego, CA, USA), PerCP-Cy5.5-anti-CD19 (BD Biosciences), BV421-anti-CD161 (BD Biosciences), APC-anti-TCRVα7.2 (BioLegend, San Diego, CA, USA), PE-CF594-anti-TCRγδ (BD Biosciences), PE-Cy7-anti-CD8α (BD Biosciences), and PE-anti-CD8β (BD Biosciences) antibodies at 4 °C for 30 min in the dark. Isotype-matched control antibodies were used as negative controls. The frequencies of various T-cell subsets were determined by flow cytometry analysis using the FACSAria II (BD Biosciences) and FlowJo software (v7.6.2; TreeStar, San Carlos, CA, USA).

### Cytometric bead array (CBA) analysis of plasma cytokines

Plasma IL-17A, TNF-α, and IFN-γ levels were determined using the CBA(Morgan et al., 2004), with minor adaptations to the manufacturer’s recommended protocol (BD Biosciences). Quantification was performed using the Cell Quest Pro software, the FACSAria II, and CBA software (BD Biosciences).

### Statistical analysis

Statistical analysis was performed using SPSS 21.0 (SPSS, Chicago, IL, USA). Quantitative data were reported as individual values or the median (range) of each group. Between-group differences were evaluated using the Kruskal-Wallis one-way analysis of variance (ANOVA) followed by the Dunn-Bonferroni post hoc method or the Mann–Whitney *U* test. Correlations were analyzed using the Spearman’s rank correlation test. Forward selection logistic regression models were used. *P*-values < 0.05 were considered statistically significant.

## Results

### Demographic and clinical characteristics of the study population

This study included 45 patients with OA and 21 matched healthy controls. Among the patients with OA, there were 22 patients with KOA and 23 patients with MOA. Demographic and clinical characteristics of the study population are summarized in**** Table 1. There were no significant differences in age, gender, BMI, or WBC count between patients with OA and healthy controls or between MOA and KOA patients. However, ESR and plasma CRP levels were significantly higher in patients with OA compared to healthy controls (*P* < 0.0001; *P* < 0.0001), and ESR was significantly elevated in patients with MOA compared to patients with KOA (*P* = 0.0021). There were no significant differences in WOMAC scores between patients with KOA and patients with MOA.

**Table 1:** Demographic and clinical characteristics of the study population.

Parameters	HC	OA		
	(*n* = 21)	Total (*n* = 45)	KOA (*n* = 22)	MOA (*n* = 23)
Age (years)	59 (52–72)	60 (37–82)	59 (37–80)	61(52–82)
Gender: female/male	13/9	26/19	13/9	13/10
BMI	22.6 (17.2–29.2)	23.1 (18–30.2)	21.05 (18.2–30.2)	23.1 (18–30.1)
ESR (mm/h)	11 (6–15)	18 (5–79)^*^	16 (5–24)^*^	21 (6–79)^*^^#^
CRP (mg/L)	2.14 (1.11–3.0)	6.5 (3.11–32.8)^*^	6.86 (3.11–29.6)^*^	5.85 (3.14–32.8)^*^
WOMAC	ND	95 (40–166)	94 (40–164)	95 (45–166)
WBC (10^9^/L)	6.16 (4.32–8.34)	5.91 (3.40–11.25)	6.24 (4-11.25)	5.88 (3.4–8.93)

**Notes.**

Data are median (range) or number of cases.

OA osteoarthritis KOA Knee-only OA MOA Multi-joint OA HC healthy controls BMI Body Mass Index ESR Erythrocyte sedimentation rate CRP C-reactive protein WOMAC The Western Ontario and McMaster Universities Osteoarthritis Index

Normal values: BMI: 18.5–24.0, ESR: 0–15 mm/h, CRP: 0–3 mg/L.

* *P* < 0.05 versus HC.

# *P* < 0.05 versus KOA.

### Increased frequencies of total MAIT cells, and MAIT cell subsets in OA

**Figure 1:**
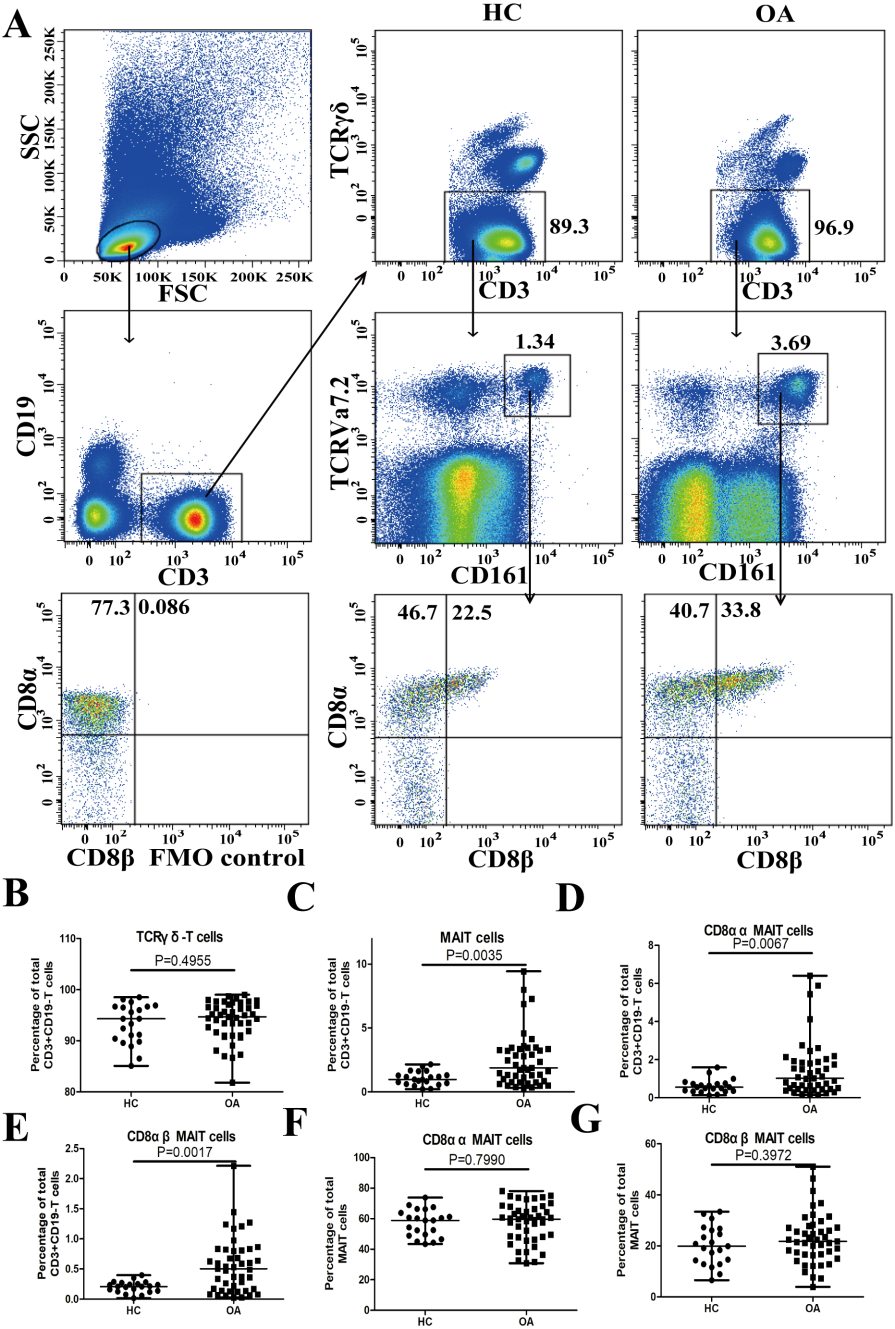
Assessment of circulating MAIT cells in patients with OA and healthy controls. PBMCs were analyzed for the percentages of TCRγδ^−^T-cells, and total, CD8αα, and CD8αβ MAIT cells by flow cytometry. (A ) Flow cytometry analysis; (B–G) quantitative analysis. Scatter plots represent mean percentage of T-cells from individual subjects. Between-group differences were analyzed using the Mann–Whitney *U* test.

MAIT cells were characterized by the high expression of CD161 and were identified by flow cytometry analysis as CD3^+^CD19^−^TCRγδ^−^*T*^high^ TCRVa7.2 and CD161^high^ (Fig. 1A). There was no significant difference in the percentage of CD3^+^CD19^−^TCRγδ cells in patients with OA and the healthy controls (Fig. 1B), but the percentage of total MAIT cells was significantly higher in patients with OA (*P* = 0.0035, Fig. 1C). The two main subgroups of MAIT cells, CD8αα and CD8αβ, were assessed using a gating strategy based on CD8α and CD8β expression (Fig. 1A). CD8αα and CD8αβ MAIT cells were identified as CD3^+^CD19^−^TCR γδ^−^TCRVa7.2^high^CD161^high^ CD8*α*^+^CD8β^−^ and CD3^+^CD19^−^TCR γδ^−^TCRVa7.2^high^CD161^high^ CD8α^+^CD8β^+^ T-cells, respectively. The percentages of CD8αα (*P* = 0.0067, Fig. 1D) and CD8αβ (*P* = 0.0017, Fig. 1E) MAIT cells were significantly higher in patients with OA compared to the healthy controls. The percentages of CD8αα (*P* = 0.7990, Fig. 1F) and CD8αβ (*P* = 0.3972, Fig. 1G) MAIT cells in the total MAIT cells were not significantly different in patients with OA compared to the healthy controls.

### Increased frequencies of total MAIT cells and MAIT cell subsets in MOA and KOA

**Figure 2:**
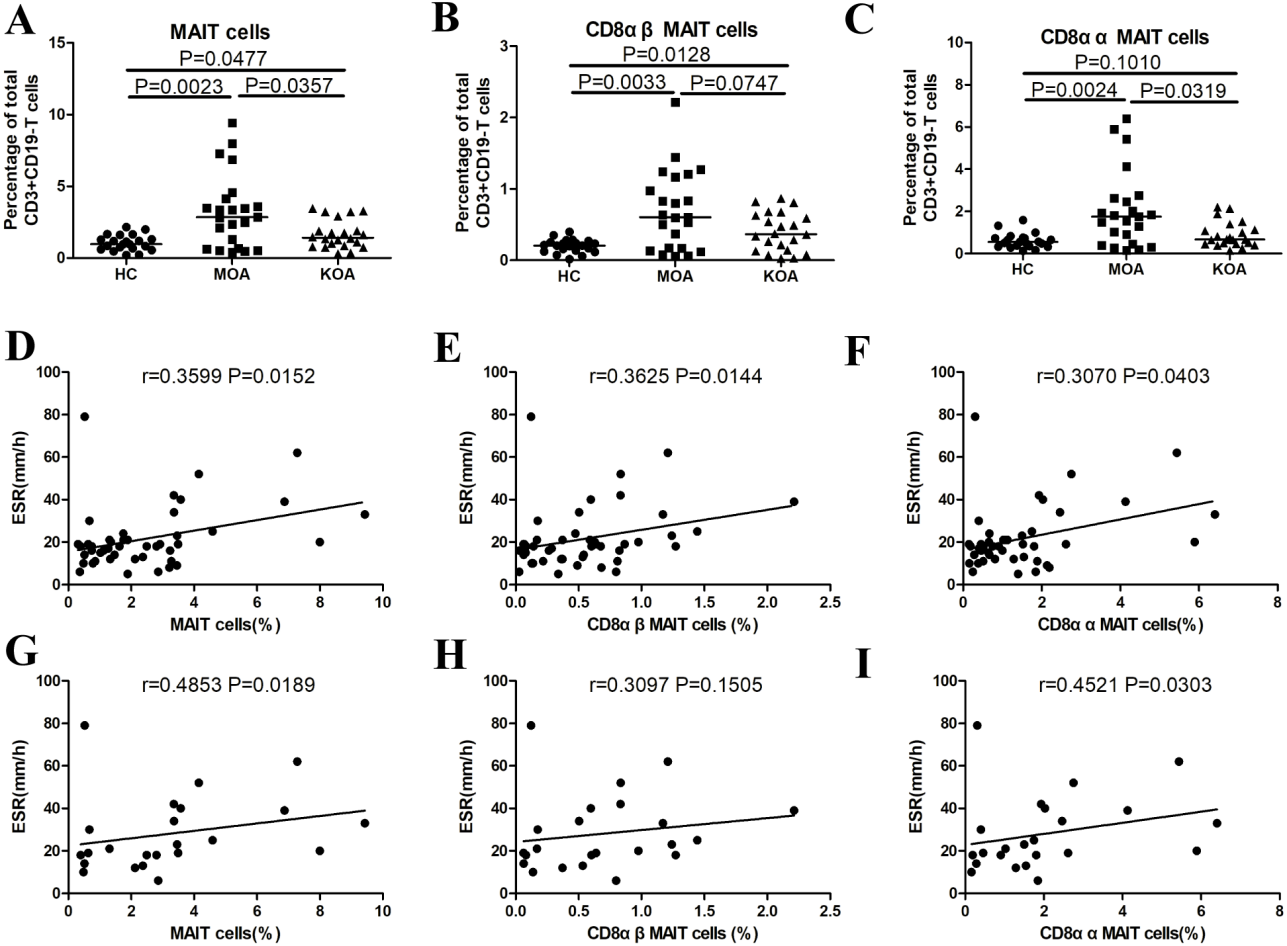
MAIT cells in patients with OA categorized by joint involvement, and correlation of MAIT cells with OA clinical parameters. (A) Percentage of total MAIT cells; (B) percentage of CD8αβ MAIT cells; (C) percentage of CD8αα MAIT cells ; (D, E and F) Correlation of total, CD8αβ and CD8αα MAIT cells with ESR in patients with OA. (G, H, and I) correlations of total, CD8αβ, and CD8αα MAIT cells with ESR in patients with MOA. Between-group differences were analyzed using the Kruskal-Wallis ANOVA followed by the Dunn-Bonferroni post hoc test. Correlation analysis was performed using the Spearman’s rank correlation test.

The percentages of total (*P* = 0.0477, Fig. 2A) and CD8αβ (*P* = 0.0128, Fig. 2B) MAIT cells were significantly higher in patients with KOA compared to the healthy controls. Similarly, the percentages of total (*P* = 0.0023, Fig. 2A), CD8αβ (*P* = 0.0033, Fig. 2B), and CD8αα (*P* = 0.0024, Fig. 2C) MAIT cells were significantly higher in patients with MOA compared to the healthy controls. The percentages of total (*P* = 0.0357, Fig. 2A) and CD8αα (*P* = 0.0319, Fig. 2C), but not CD8αβ (Fig. 2B), MAIT cells were significantly higher in patients with MOA compared to patients with KOA.

### Correlation of MAIT cells and clinic indicators of OA

There were significant positive correlations between the percentages of total (*P* = 0.0152, *r* = 0.3599; Fig. 2D), CD8αβ (*P* = 0.0144, *r* = 0.3625; Fig. 2E) and CD8αα (*P* = 0.0403 *r* = 0.3070; Fig. 2F) MAIT cells and ESR in patients with OA. There were significant positive correlations between the percentages of total (*P* = 0.0189, *r* = 0.4853; Fig. 2G) and CD8αα (*P* = 0.0303, *r* = 0.4521; Fig. 2I) MAIT cells and ESR in patients with MOA. There were significant positive correlations between the percentages of total (*P* < 0.05) and CD8αα (*P* < 0.05) MAIT cells and WOMAC scores in patients with OA and MOA (Table 2). There was a significant positive correlation between the percentage of CD8αβ MAIT cells and WOMAC scores in patients with MOA (Table 2). There were no significant correlations between the percentages of total, CD8αα, or CD8αβ MAIT cells and WOMAC scores in patients with KOA.

**Table 2:** Correlation of MAIT cell populations (% CD3^+^CD19^−^ T-cells) with WOMAC scores.

MAIT Cell Populations	OA		KOA		MOA	
	r	*P*	r	*P*	r	*P*
Total	0.2944	0.0496^*^	−0.0102	0.9642	0.4421	0.0346^*^
CD8αα	0.3162	0.0343^*^	0.0774	0.7321	0.4520	0.0304^*^
CD8αβ	0.2677	0.0755	−0.0650	0.7740	0.5069	0.0136^*^

**Notes.**

WOMAC The Western Ontario and McMaster Universities Osteoarthritis Index OA osteoarthritis KOA Knee-only OA MOA Multi-joint OA

All analyses were performed using Spearman’s rank correlation test.

* *P* < 0.05.

### Correlation of MAIT cells and plasma IFN-γ levels in OA

**Figure 3:**
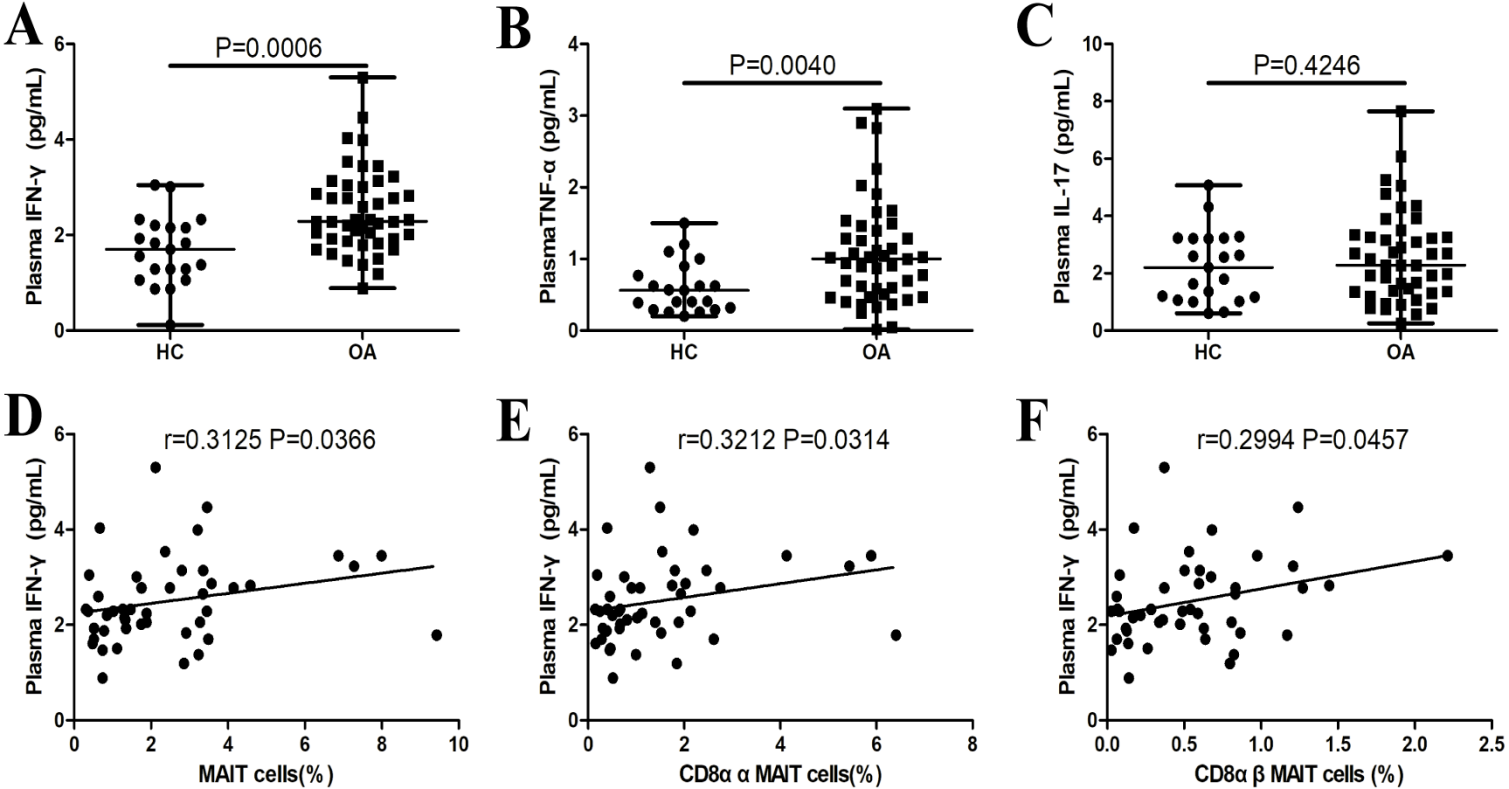
Plasma IFN-γ, TNF-α, and IL-17 levels in patients with OA. (A) IFN-γ; (B) TNF-α; (C) IL-17; (D, E, and F) correlations between plasma IFN-γ level and the percentages of total, CD8αα, and CD8αβ MAIT cells in patients with OA. Between-group differences were analyzed using the Mann–Whitney *U* test. Correlation analysis was performed using the Spearman’s rank correlation test.

Plasma IFN-γ (*P* = 0.0006, Fig. 3A) and TNF-α (*P* = 0.0040, Fig. 3B) levels were significantly higher in patients with OA compared to the healthy controls. There were significant positive correlations between plasma IFN-γ levels and the percentages of total (*P* = 0.0366, *r* = 0.3125; Fig. 3D), CD8αα (*P* = 0.0314 *r* = 0.3212; Fig. 3E), and CD8αβ (*P* = 0.0457 *r* = 0.2994; Fig. 3F) MAIT cells in patients with OA. There were no significant correlations between plasma TNF-α or IL-17 levels and the percentages of total, CD8αα, or CD8αβ MAIT cells in patients with OA.

### Plasma IFN-γ, TNF-α, and IL-17 levels in patients with MOA and KOA compared to healthy controls

**Figure 4:**
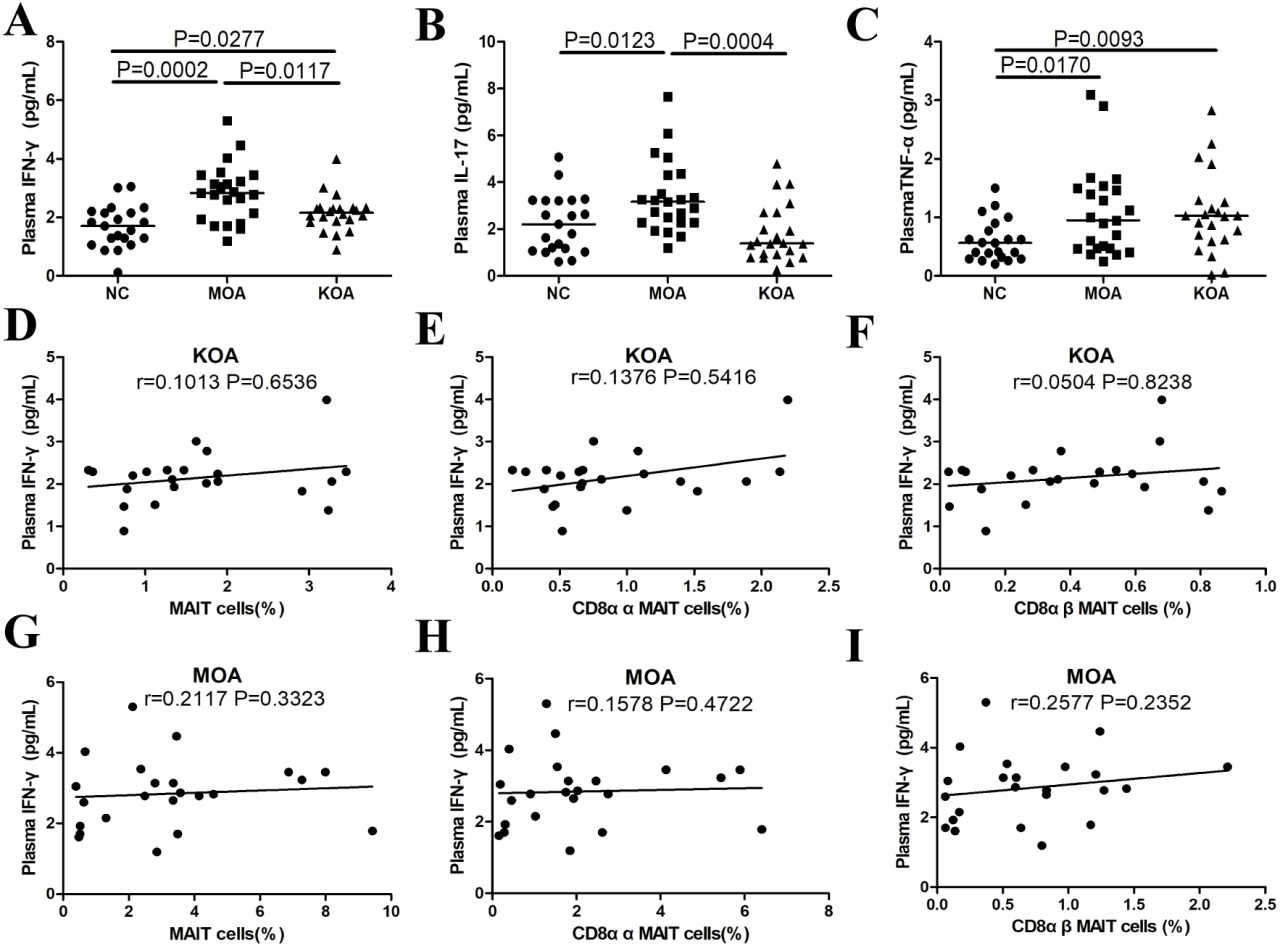
Plasma cytokine levels in patients with OA categorized by joint impairment, and correlation analysis of plasma cytokine levels with MAIT cells. (A) IFN-γ ; (B) TNF-α; (C) IL-17. (D–I) Correlations of plasma IFN-γ level with total, CD8αα, and CD8αβ MAIT cells in patients with KOA and MOA. Between-group differences were analyzed using the Kruskal–Wallis ANOVA followed by the Dunn-Bonferroni post hoc test. Correlation analysis was performed using the Spearman’s rank correlation test.

Plasma IFN-γ levels were significantly higher in patients with KOA (*P* = 0.0277, Fig. 4A) and MOA (*P* = 0.0002, Fig. 4A) compared to the healthy controls. Plasma TNF-α levels were significantly higher in patients with KOA (*P* = 0.0093, Fig. 4B) and MOA (*P* = 0.0170, Fig. 4B) compared to the healthy controls. Plasma IL-17 levels were significantly higher in patients with MOA (*P* = 0.0004, Fig. 4C) compared to the healthy controls. Plasma IFN-γ (*P* = 0.0117, Fig. 4A) and IL-17 (*P* = 0.0123, Fig. 4C) levels were significantly higher in patients with MOA compared to patients with KOA, but there was no difference in plasma TNF-α levels between the two groups. There was no correlation between plasma cytokine levels and MAIT cell subsets in patients with MOA or KOA (Figs. 4C–4I and Fig. S1).

### Binary logistic regression analysis shown the frequency of MAIT cells was associated with the risk of OA

With OA patients as the dependent variable (OA = 1, health control=0) and the frequency of total MAIT cell subsets and plasma cytokine levels as independent variables, a binary logistic regression model, were established. The Hosmer-Lemeshow test was conducted to assess the goodness of fit of the model and the *P*-value of 0.872 indicated a good fit. After adjusting for the levels of plasma cytokines (IFN-γ and TNF-α), the frequency of MAIT cells was associated with the risk of OA (*P* = 0.019, OR =3.121, 95% CI [1.206–8.077]), as shown in Table 3.

**Table 3:** Variables in the equation.

		B	S.E.	Wald	df	Sig.	Exp(B)	95.0% C.I. for Exp (B)	
								Lower	Upper
Step 4^*a*^	IFN-γ	1.587	0.66	5.787	1	0.016	4.891	1.342	17.829
	TNF-α	1.877	0.774	5.882	1	0.015	6.537	1.434	29.804
	MAIT	1.138	0.485	5.501	1	0.019	3.121	1.206	8.077
	Constant	−5.449	1.796	9.205	1	0.002	0.004		

## Discussion

This study investigated the potential role of MAIT cells in OA by determining the percentages of total, CD8αα, and CD8αβ MAIT cells, along with plasma IFN-γ, TNF-α, and IL-17 levels, and their associations with clinical parameters and disease severity in patients with OA. The findings showed a higher percentage of total MAIT cells in patients with OA compared to the healthy controls. In accordance with these data, a previous study revealed that MAIT cells enhanced inflammation and exacerbated collagen-induced arthritis in a murine model of multiple sclerosis (Chiba et al., 2012). Specifically, the current study identified an increasing percentage of CD8αα and CD8αβ MAIT cells in patients with OA compared to healthy controls, and a significant positive correlation between the percentages of total, CD8αα, and CD8αβ MAIT cells with ESR and WOMAC scores, in this patient population. Binary logistic regression analysis showed that the frequency of MAIT cells was associated with the risk of OA. To our knowledge, the current study is the first to explore the frequencies of total and MAIT cell subsets in patients with OA, and to implicate MAIT cells and their subpopulations as potential biological markers of OA disease severity.

The current study showed that patients with OA had higher plasma IFN-γ and TNF-α levels compared to healthy controls, and the percentages of total, CD8αα, and CD8αβ MAIT cells were positively correlated with IFN-γ levels in patients with OA. Previous reports indicated that MAIT cells express *ROR*γ*t* and *T-bet* transcription factors and have the ability to secrete proinflammatory cytokines, including IL-17, IFN-γ, and TNF-α (Kawachi et al., 2006; Dusseaux et al., 2011), and that IFN-γ, IL-17, and TNF-α promote abnormal remodeling of joint tissues in OA (Shen et al., 2011; Wojdasiewicz, Poniatowski & Szukiewicz, 2014). Taken together, these findings suggest that MAIT cells are associated with inflammation. The level of plasma IFN-γ was positively correlated with the frequency of MAIT cells, which may be related to the secretion of IFN-γ by MAIT cells, or MAIT cells responding to IFN-γ as a chemotactic cue. The specific underlying mechanisms require further study.

To inform treatment decisions in OA, the OA Research Society International (OARSI) guidelines for the non-surgical management of knee osteoarthritis defined two clinical sub-phenotypes of knee OA according to OA joint type, including KOA and MOA (McAlindon et al., 2014). In the current study, patients with MOA had higher percentages of total, CD8αβ, and CD8αα MAIT cells compared to healthy controls, and there were positive correlations between total and CD8αα MAIT cells with ESR and WOMAC scores. These results describe the frequencies of total and MAIT cell subsets in patients with MOA, but also implicate MAIT cells and their subpopulations as potential biological markers of MOA disease severity.

In the current study, the percentage of CD8αα MAIT cells was higher in patients with MOA compared to those with KOA or the healthy controls. Recent evidence suggests that CD8αα and CD8αβ MAIT cells have similar effector functions (IFN-γ, IL-17, IL-22) and chemokine receptor expression (CCR2, CCR6, CXCR6), and that CD8αα MAIT cells may originate from CD8αβ MAIT cells. In a mouse model of T-cell mediated autoimmune disease, CD8αα MAIT cells were involved in a negative feedback regulatory mechanism that limited uncontrolled T-cell expansion (Tang et al., 2006). In humans, CD8αα MAIT cells isolated from adult PMBCs secreted more IL-17A after stimulation with PMA/ionomycin than CD8αβ cells, suggesting an active role in immunity and tissue inflammation, possibly as tissue infiltrates in inflammatory arthritis or the liver in hepatitis C infection (Walker et al., 2012). In this study, plasma IL-17 and IFN-γ levels were higher in patients with MOA compared to those with KOA or the healthy controls, consistent with the elevation of CD8αα MAIT cells in patients with MOA. These observations indicate that MOA is a clinical sub-phenotype of OA, with characteristic inflammatory features. Evidence suggests that patients with inflammatory OA have a poor prognosis (Sellam & Berenbaum, 2010), and involvement of multiple joints is an important prognostic predictor of the progression of KOA (Bastick et al., 2015). Interestingly, several studies have reported a selective decrease in the percentage of MAIT cells in patients with systemic lupus erythematosus or RA. However, these findings should be interpreted with caution as 90% of patients included in these studies were receiving corticosteroids. In the current study, patients who had received glucocorticoids were excluded, as corticosteroids are known to decrease the proliferation and function of MAIT cells (Hinks, 2016).

This study was associated with some limitations. First, MAIT cells were only detected in peripheral blood samples and the infiltration of MAIT cells into the joints was not investigated. Second, this was a small cross-sectional study. Third, MR1 tetramers may allow more accurate identification of MAIT cells than Vα7.2 and CD161 expression because of variability in CD161 expression. However, human MR1-restricted T cells can be divided into typical MAIT cells and atypical MR1-restricted T cells, and diversity within the MR1-restricted T cell repertoire leads to differing MR1-restricted Ag specificity (Gherardin et al., 2016; D’Souza, Chen & Corbett, 2018). As a typical antigen of MAIT cells, 5-OP-RU is routinely used to produce MR1-Ag tetramers, which effectively stain a large majority of MR1-restricted cells, but not all (Gherardin et al., 2016; D’Souza, Chen & Corbett, 2018). In future, with increased understanding of other MR1-restricted T cells and MR1-restricted Ag, MR1-restricted recognition will enable more accurate identification of MAIT cells. We will further explore the mechanistic role of MAIT cells by defining them with MR1 tetramers in the synovial fluid or tissues of a large sample of patients with OA at multiple time points.

## Conclusions

MAIT cells and their subpopulations were significantly increased in patients with OA and have potential as biological markers of OA disease severity, especially in patients with MOA. These findings add to the evidence base describing the changes in immune cells in OA and the immunological differences between MOA and KOA.

##  Supplemental Information

10.7717/peerj.7443/supp-1 Supplemental Information 1 Raw data In the sequence “multijoint”, 1 represents patients with KOA, 2 represents patients with MOA, and 0 represents healthy control. “TCR *γ δ* , MAIT, CD8AA and CD8AB” respectively refer to the proportion of TCR *γδ*^−^, MAIT, CD8αα, CD8αβ cells in CD3^+^CD19^−^ T cells, while “CD8αα and CD8αβ” respectively refer to the proportion of CD8αα, CD8αβ cells in MAIT cells.

10.7717/peerj.7443/supp-2 Supplemental Information 2 Correlation analysis of plasma cytokine levels with MAIT cells **A**, **B**, **C**, **D**, **E**, and** F**: Correlations of plasma IL-17 level with total, CD8αα, and CD8αβ MAIT cells in patients with KOA and MOA. **G**, **H**, **I**, **J**, **K**, and** L**: Correlations of plasma TNF- level with total, CD8αα, and CD8αβ MAIT cells in patients with KOA and MOA. Correlation analysis was performed using the Spearman’s rank correlation test.

## Additional Information and Declarations

### Data Availability

The following information was supplied regarding data availability: The raw data is available as a Supplemental File.
