# Supplementary figures and images for: Elevated frequencies of total and MAIT cell subsets in patients with knee osteoarthritis
Source: PeerJ. 2019 Aug 5;7:e7443. doi: 10.7717/peerj.7443 (PMC6686836; doi:10.7717/peerj.7443)

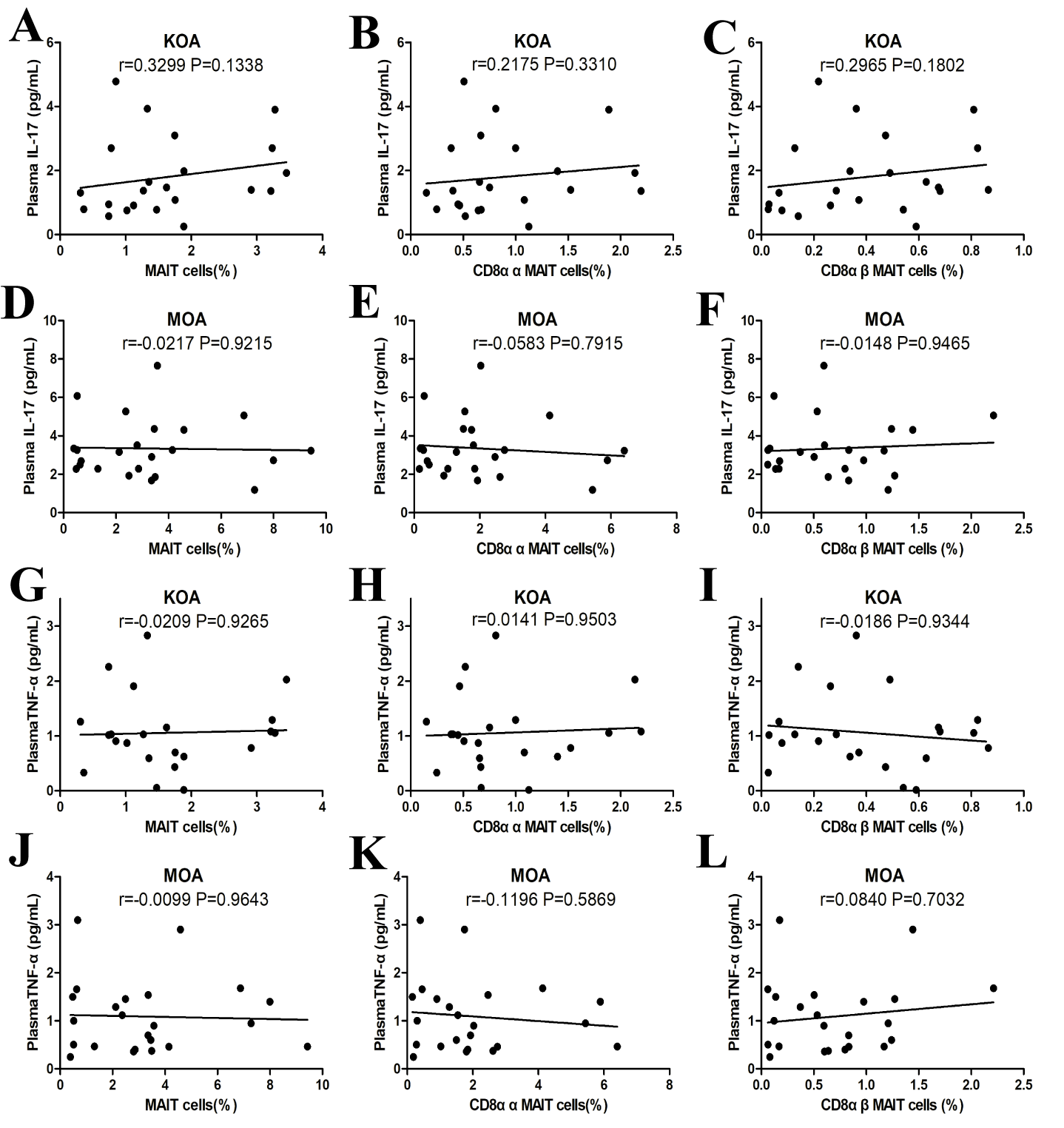

Supplement: Supplemental Information 2 — A, B, C, D, E, and F: Correlations of plasma IL-17 level with total, CD8αα, and CD8αβ MAIT cells in patients with KOA and MOA. G, H, I, J, K, and L: Correlations of plasma TNF- level with total, CD8αα, and CD8αβ MAIT cells in patients with KOA and MOA. Correlation analysis was performed using the Spearman’s rank correlation test. [file peerj-07-7443-s002.png]
